# Supplementary figures and images for: Destruction of the vascular viral receptor in infectious salmon anaemia provides in vivo evidence of homologous attachment interference
Source: PLoS Pathog. 2022 Oct 14;18(10):e1010905. doi: 10.1371/journal.ppat.1010905 (PMC9621750; doi:10.1371/journal.ppat.1010905)

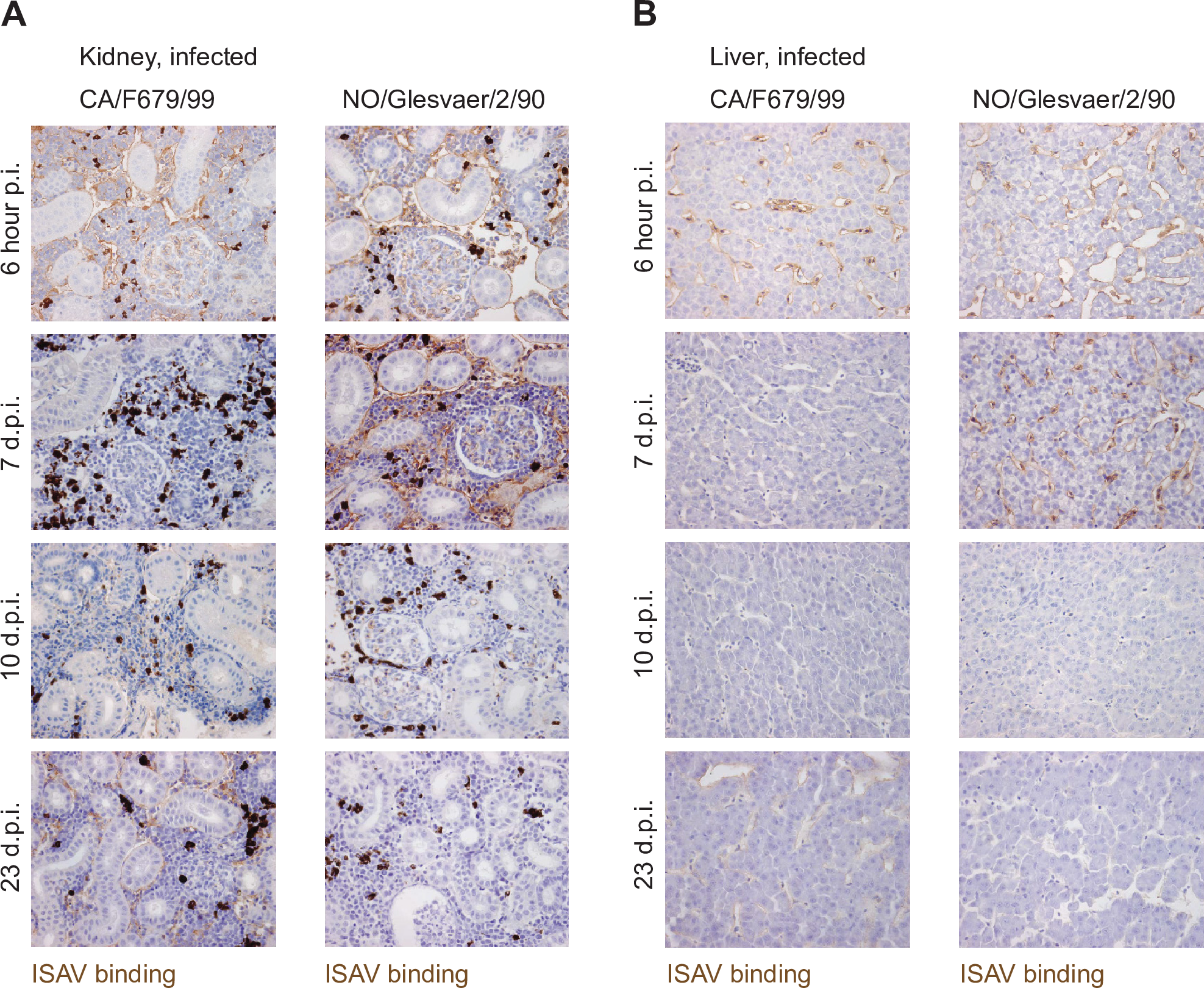

Supplement: S1 Fig — Representative micrographs of virus histochemistry in sections from kidney (A) and liver (B) of fish infected with CA/F679/99 and NO/Glesvaer/2/90, respectively. (n = 1 fish per time group). Positive binding is identified by DAB (ISAV binding, light brown). The dark brown cellular signal in kidneys is pigment in melanomacrophages and should not be confused with positive signal. (TIF) [file ppat.1010905.s001.tif]

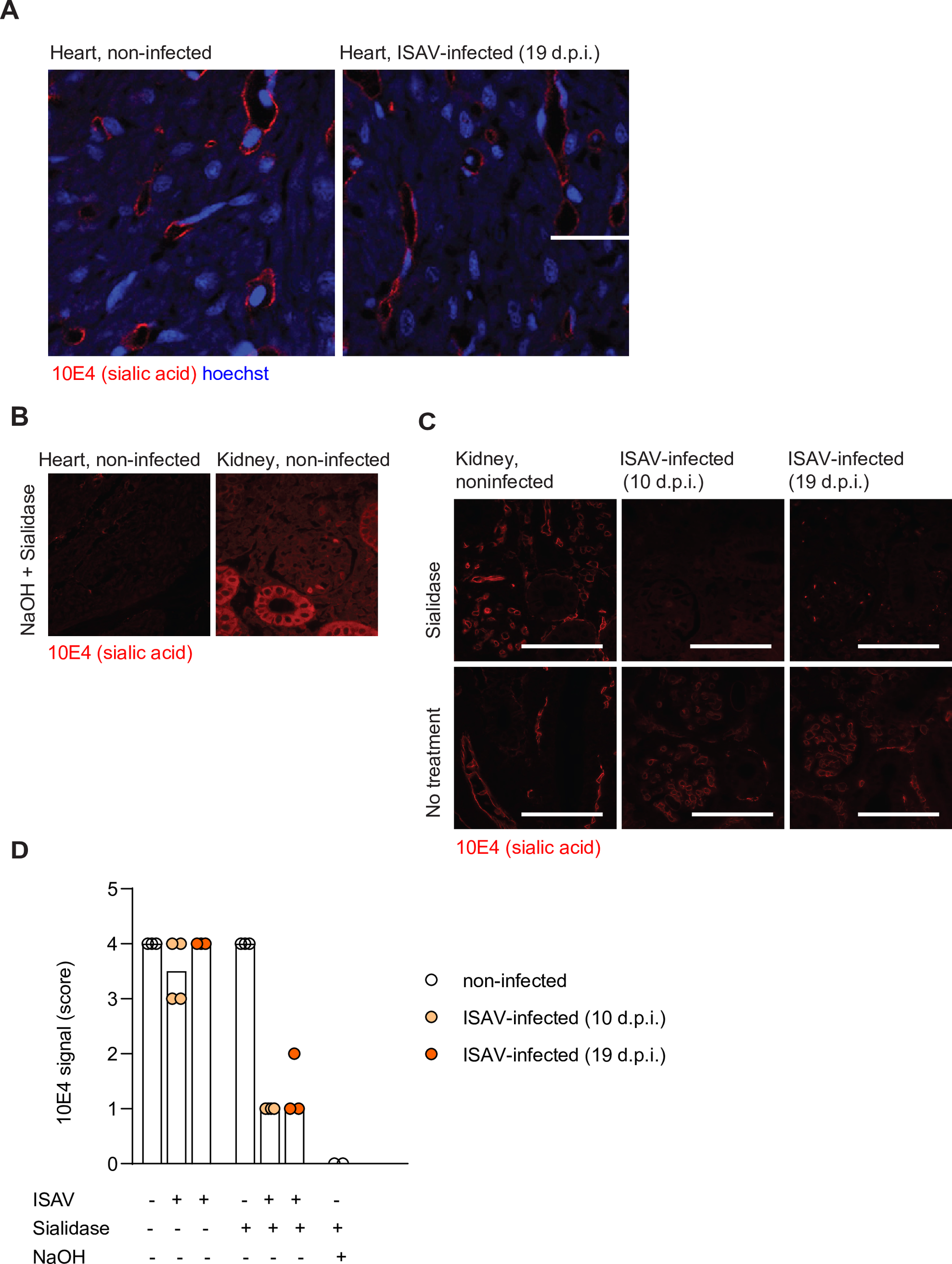

Supplement: S2 Fig — Representative micrographs of 10E4 immunofluorescent staining of sialic acid (red). (A) 10E4 staining of hearts of non-infected and NO/Glesvaer/2/90 infected (19 d.p.i.) fish showed no difference in signal intensity. (B) No 10E4 signal could be detected in tissue sections of non-infected fish pre-treated with NaOH and sialidase (negative control for Figs 2B and S2C). The extensive autofluorescence in kidney tubules should not be confused with positive signal. (C-D) 10E4 signal in kidney sections of experimentally infected and non-infected fish (n = 4 fish per group), confirming findings from heart, that NO/Glesvaer/2/90 infection renders the 10E4 epitope sensitive to sialidase. (TIF) [file ppat.1010905.s002.tif]

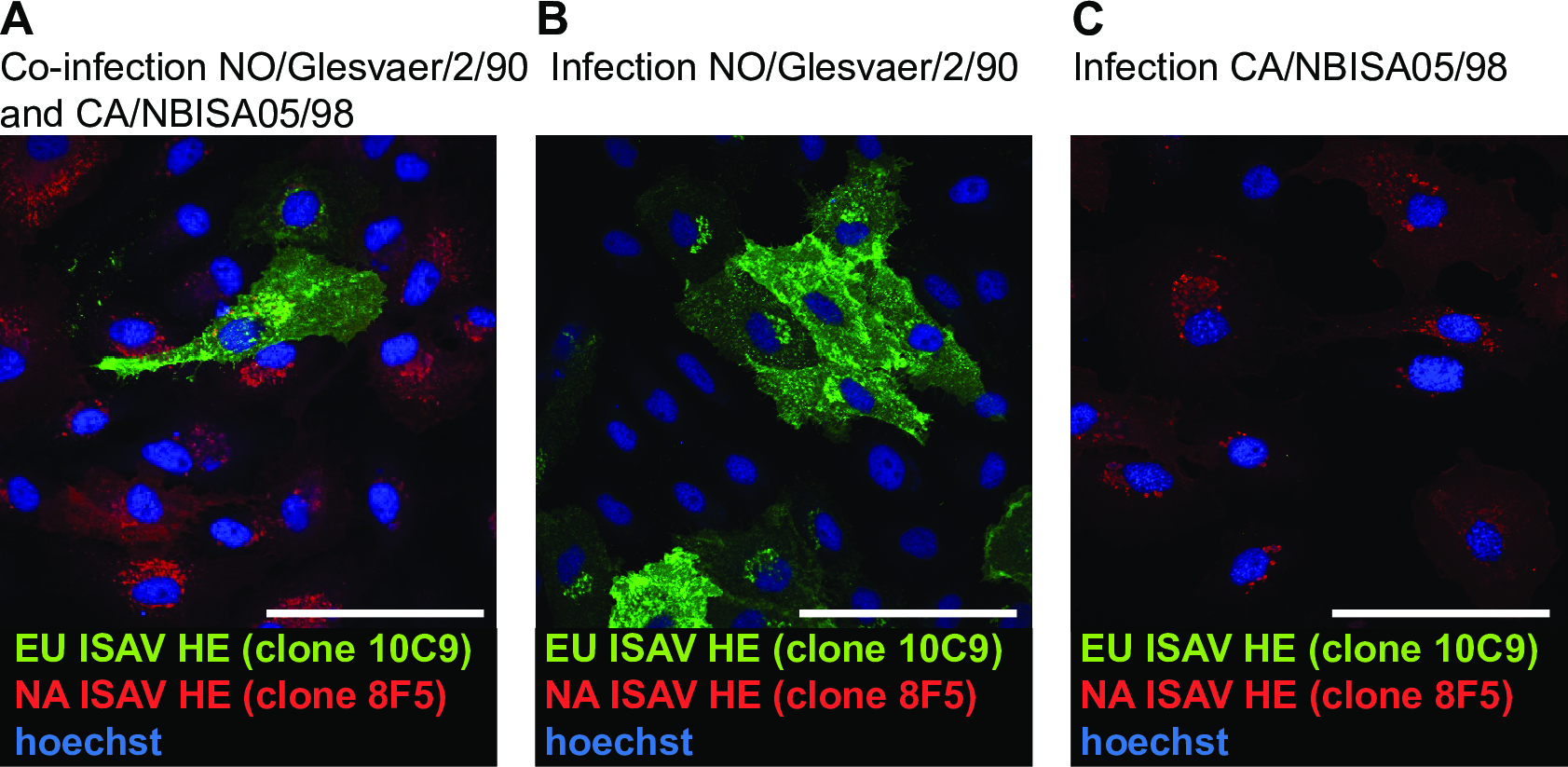

Supplement: S3 Fig — (A) NO/Glesvaer/2/90 and CA/NBISA05/98 co-infected ASK cells, (B) NO/Glesvaer/2/90 infected ASK cells, and (C) CA/NBISA05/98 infected ASK cells were fixed and immunostained with the hemagglutinin esterase (HE)-reactive antibody clones 10C9 (green) and 8F5 (red), specific to European and North-American ISAV genogroups, respectively. (TIF) [file ppat.1010905.s003.tif]
