# Supplementary material for: Destruction of the vascular viral receptor in infectious salmon anaemia provides in vivo evidence of homologous attachment interference
Source: PLoS Pathog. 2022 Oct 14;18(10):e1010905. doi: 10.1371/journal.ppat.1010905 (PMC9621750; doi:10.1371/journal.ppat.1010905)
Supplement: S1 Table — (DOCX) [file ppat.1010905.s004.docx]

## Table S1. Reagents and antibodies

| Type of reagent | Product name | Source  (catalog number) | Comment |
| --- | --- | --- | --- |
| Cell culture medium | L-15 | Lonza  (12-700F) | Supplemented with FBS, L-glutamine, and penicillin/streptomycin/ amphotericin, as detailed below. |
| Supplement | Fetal bovine serum | Lonza  (DE14-801F,  lot. 0SB015) | Used 1/10 (10%) |
| Supplement | L-glutamine | Lonza (17-605E) | Used 1/50 = 4 mM |
| Antimicrobial supplement | Penicillin/Streptomycin/ amphotericin | Lonza (17-745E) | Used 1/100 |
| Antibody (1°) | Mouse IgG1 anti-ISAV HE (clone 3H6F8) | Knut Falk, Norwegian Veterinary Institute | Each batch of hybridoma supernatants was titered before use. Typically, supernatants were used at 1/10-1/100 dilution in IF, VBA and CELISA. |
| Antibody (1°) | Mouse IgG1 anti-ISAV HE (clone 10C9, specific to European ISAV genogroups) | Knut Falk, Norwegian Veterinary Institute | Hybridoma supernatant was used 1/10 for strain-specific detection of ISAV HE in IF, VBA and CELISA. |
| Antibody (1°) | Mouse IgG2b anti-ISAV HE (clone 8F5, specific to North-American ISAV genogroups) | Sharon Clouthier, Fisheries and Oceans Canada | Hybridoma supernatant was used 1/10-30 for strain-specific detection of ISAV HE in IF, VBA and CELISA. |
| Antibody (1°) | Mouse IgG1 anti-ISAV NP (clone NP10) | Aquatic Diagnostics (P10) | Used 1/500 |
| Antibody (1°) | Mouse anti-IHNV N protein (clone 136-3) | Niels Jørgen Olesen, EURL for fish and crustacean disease, DTU, Denmark | Hybridoma supernatant was used at 1/50 dilution in IF and CELISA |
| Antibody (1°) | Mouse IgM detecting the backbone of the cellular ISAV receptor (clone 10E4) | Knut Falk, Norwegian Veterinary Institute | Hybridoma supernatant was used 1/100 in IF and IHC |
| Antibody (1°) | Rabbit anti-ISAV NP reactive serum (K716) | Knut Falk, Norwegian Veterinary Institute | Used 1/3000 for IHC |
| Antibody (2°) | Goat anti-mouse IgG - HRP | Invitrogen (UK292754) | Used 1/5000 |
| Antibody (2°) | Goat anti-mouse IgM – Alexa594 | Molecular probes (A21044) | Used 1/200 |
| Antibody (2°) | Goat anti-mouse IgG (H+L) – Alexa488 | Molecular probes (A11001) | Used 1/500 |
| Cell marker | Phalloidin Alexa594 | Molecular probes (A12381) | Used 1/100 |
| Cell marker | Hoechst 33342 | Thermo Fisher Scientific (H3570) | 2 µg/mL |
| Substrate | TMB | Thermo Fisher Scientific (VD301152) | Used undiluted |
| Blocking buffer | 10x clear milk block (CMB) | Pierce (UL2891131) | Used x1 for VBA and CELISA |
| IHC detection system | EnVision Peroxidase/DAB, rabbit/mouse | Agilent DAKO | Used for VHC and IHC |
| IHC detection system | MACH2 Universal HRP-Polymer Detection | Biocare (M2U522G) | Used for VHC |
| IHC detection system | DAB Chromogen kit | Biocare  (DB801L) | Used with MACH2 for VHC |
| Blocking buffer | Background sniper | Biocare (BS966H) | Used with MACH2 for VHC |
| Antibody diluent | Da Vinci Green Diluent | Biocare (PD900L) | Used with MACH2 for VHC |

IF = immunofluorescent staining; VBA= virus binding assay, CELISA = cell-based enzyme-linked immunoassay; IHC = immunohistochemistry
