# Supplementary material for: Destruction of the vascular viral receptor in infectious salmon anaemia provides in vivo evidence of homologous attachment interference
Source: PLoS Pathog. 2022 Oct 14;18(10):e1010905. doi: 10.1371/journal.ppat.1010905 (PMC9621750; doi:10.1371/journal.ppat.1010905)
Supplement: Attachment — Submitted filename: Point-to-point_PP2022.docx [file ppat.1010905.s006.docx]

## Point to point response to the reviewers of our manuscript: "Destruction of the vascular viral receptor in infectious salmon anaemia provides in vivo evidence of homologous attachment interference"

For clarity, we have included the original comments from the reviewers in black and inserted our response to each comment in blue below.

**Part I - Summary**
Please use this section to discuss strengths/weaknesses of study, novelty/significance, general execution and scholarship.

**Reviewer #1:** The manuscript by Maria Aamelfot describes that ISAV infection removes viral receptors that as such the fish become refractory to a subsequent infection. ISAV is a member of the orthomyxoviridea carrying an HE and fusion envelope protein. The HE protein is special as it binds and cleaves 4-0-Ac sialic acids. I my knowledge only MHV HE has a similar specificity and activity (not referenced). Especially nice is the demonstration that a heterologous virus infection does not inhibit ISAV infection.

The manuscript is of great interest and I for sure would recommend it for publication in Plos Path and only have several suggestions to improve the paper.

**Response:** Thank you for the thorough work of reviewing our manuscript and for the many constructive comments. We have responded to each comment in turn below in blue colour. We have also cited the references showing that the receptor binding and destruction of puffinosis coronavirus and MHV is specific to 4-*O*-acetylated sialic acids (Ref 10: Langereis et al, 2010; Ref 11: Regl et al, 1999; Ref 12: Klausegger et al, 1999).

Although the figures are presented nicely, they could be improved to follow them without text. Especially from fig 4 and the supplemental in which it is hard to follow what is stained (which tissue / which antibody specific for x?) and what the treatment was, for most readers VHC, RBC IFAT, and VBA block become a puzzle. I’m also not such a fan of the different scoring systems, why is this not quantifiable? Panel E is interesting but counterintuitive as 1% means an increase and the other a decrease. Figure 4 could thus use an overhaul.

**Response:** Thank you for bringing this to our attention.

We have revised figures 1, 4, and S2 with improved labelling and by plotting residual binding rather than % inhibition, and hope the figures are now easier to read. We have also improved the labelling of micrographs in figures 2, 5, and S1-S3.

We agree that an objective and quantitative system for measuring signal would be better and perhaps give a somewhat better resolution. We are in the process of setting this up for future infection trials, i.e. scanning of sections labelled by immunohistochemistry and virus histochemistry followed by digital analysis, and flow cytometry to assess the ISAV-coating of red blood cells. However, the complete validation of the digital analysis technique is still pending in our lab; and waiting for this, in addition to new rounds of staining and analyses, would significantly delay the resubmission of the manuscript. Moreover, we do not have preserved red blood cells from this trial to reassess the ISAV-coating by flow cytometry. Because pilots suggest that the results of digital analyses correspond well to the previously used manual scoring systems, together with the marked and very consistent nature of the change, we are confident that digital analyses would not change the results of the current study. We therefore hope it is acceptable to present the data in their current format.

The authors nicely reference old-school Schauer literature, but recent literature is dotted with gems describing specific 0-acetyl binding by HE and spike.

Imho the introduction and discussion should be condensed to make it easier to follow and a quicker read. I understand the fact that a homologous infection prevents a secondary by removing a particular sialic acid is of interest, but it is a bit of an open door as well. The discussion goes from one topic to the other and back. I would like to read something more focused on why this 4-0Ac sialic acid is of such interest as viral receptors. For example horse a-macroglobulin is extremely rich in 4-OAc and historically used as an inhibitor (with very high concentrations) for influenza A viruses, would this now be a perfect inhibitor for ISAV?

**Response:** Thanks for the feedback to the introduction and discussion, which was of great help to try to improve the readability of these sections. We have condensed the introduction and included several recent examples of specific *O*-acetyl binding, as well as a more thorough description of the early characterisation of ISAV – receptor interactions: In these publications, horse serum proteins (as well as rabbit and guinea-pig serum proteins) were indeed demonstrated to inhibit ISAV binding to red blood cells, and we have mentioned this in the introduction. We have also condensed the discussion and included subheadings to make this section easier to read.

I know this is hard but are there no specific lectins that only bind 4-0Ac? The 10e4 antibody does not depend on the 4-0Acetyl, and fig 2B top middle panel gives a distinct binding pattern? An ISAV HE-fc with enzymatic knockout would be a great way to go (or MHV?).

**Response:** Thanks for this suggestion. To our knowledge, the only 4-*O*-acetyl-sialic acid-binding lectin is the cancer antennarius lectin. However, this lectin is not completely specific, as it also binds 9-*O*-acetyl-sialic acids. We therefore followed your suggestion of using recombinant ISAV HE as a probe. The results confirmed the loss of 4-*O*-acetylation and have been included in figure 2 (Fig 2A).

**Reviewer #2:** This study by Aamelfot, Hol Fosse and colleagues examines the broadly important question of viral superinfection exclusion (ie, how a virally infected cell is able to prevent subsequent infection by a homologous virus) using infectious salmon anemia virus (ISAV). The authors show that this resistance to infection follows the de-acetylation of sialic acids on the cell surface, in the relevant animal model during the full course of infection/disease. The destruction of the viral surface receptor was able to prevent re-infection with a related ISAV strain, and the authors demonstrated that this interference was specific to ISAV (as prior infection with either a different RNA virus, IHNV, or pre-treatment with Poly I:C was not able to prevent ISAV infection). The question of how and why virally-infected cells are able to prevent superinfection by homologous virions has important implications for the field and this study provides a valuable look at how this process occurs for a segmented RNA virus in the in vivo host.

**Response:** Thank you for the thorough work you have invested in reviewing our manuscript and for the interesting experiments you have suggested. We have responded to each comment in turn in blue.

It would be very interesting to see the direct effect of transfecting HE (or more specifically the esterase portion) on the stability of the viral receptor, and whether the downregulation observed in infection occurs in the context of this protein alone.

**Response:** Thank you for this suggestion. We would very much like to follow this approach, but have not yet been able to establish protocols that allow us to transfect ASK cells with satisfactory viability and transfection efficiency. While it should be possible to overcome this obstacle, and we hope to do so in the future, we expect it will require considerable time and effort and require further funding, that we indeed will apply for as a continuation of the work described in the current manuscript

As an alternative approach to your suggestion, we tried to use another fish cell line that is more amenable to transfection: the CHSE-214 cell line. Unfortunately, and despite reported permissiveness to North-American ISAV strains by one publication (DOI 10.1099/0022-1317-81-1-143), we were unable to detect robust virus binding in non-transfected cells of this cell line. In this context, it is worth noting that CHSE-214 cell lines from different labs in our hands behave very differently, and previous efforts by us to infect several of the available CHSE-214 lab-lines with ISAV (both European and North-American) have been unsuccessful, suggesting that the permissiveness reported above could be limited to some lab-lines only.

The authors elegantly look at receptor degradation in the context of days-to-weeks, over the entire course of infection. It would be very interesting to look at the kinetics of receptor loss early after infection as well, to determine the first time at which degradation/superinfection exclusion occurs (and whether the two happen at the same time over the very early times of infection or if there is a separation in relative kinetics that suggests multiple mechanisms of SIE).

**Response:** Thank you for this suggestion. We agree that this is a very interesting question. Nevertheless, as our virus binding assays do not robustly detect inhibition of ISAV binding at earlier time points than those shown in fig 4A-B and 4E (6 d.p.i. in infected fish and 24 h in cell culture, respectively), it has been difficult to follow this line of investigation. Similarly, detection of viral HE in ASK cell cultures by immunostaining before 24 hours is very limited (only some Golgi staining is visible at 12 hours).

Reviewer #3: PPATHOGENS-D-22-01074

Destruction of the vascular viral receptor in infectious salmon anaemia provides in vivo
evidence of homologous attachment interference

In this ms the authors have investigated the possible role of ISAV hemagglutinin esterase (HE) as a mediator of viral interference in Atlantic salmon infected with ISAV. Viral interference (one infection inhibits another) is an understudied phenomenon in virology and certainly regarding fish viruses. The authors have developed a virus histochemistry method facilitating the quantification of tissue attachment sites for ISAV by measuring binding of virus to tissue sections. Using this and complementary methods the authors test the hypothesis that when ISAV infects endothelial cells of Atlantic salmon, it restricts subsequent homologous infection by removing the 4-O-acetyl group necessary for binding from sialic acid. Interesting differences in this activity between high and low virulent isolates of ISAV is measured and discussed in relation to pathogenesis. The loss of viral receptors upon infection reduce coinfection with other strains and limit the extent of host damage.

This study is well planned and conducted with relevant controls and sufficient replications. The results are presented in clear figures with appropriate statistics.
The presented data supports the authors claims

It is certainly of interest to virologists in general and of special interest to fish disease scientists.

**Response:** Thank you for the thorough work you have invested in reviewing our manuscript, your kind enthusiasm for our manuscript, and for pinpointing issues that should be improved. We have responded to the points raised under “minor issues” in turn in blue.

**Part II – Major Issues: Key Experiments Required for Acceptance**
Please use this section to detail the key new experiments or modifications of existing experiments that should be absolutely required to validate study conclusions.

Generally, there should be no more than 3 such required experiments or major modifications for a "Major Revision" recommendation. If more than 3 experiments are necessary to validate the study conclusions, then you are encouraged to recommend "Reject".

Reviewer #1: none

Reviewer #2: I think the conclusions of the study are well supported by the data. I think the paper would be strengthened if the authors included slightly more data tying the phenomenon directly to HE protein expression, and/or mapping the induction of SIE vs receptor loss at a finer level of kinetic resolution early in infection in cultured cells.

**Response:** Thank you for these suggestions. We agree that it would strengthen the paper to include such data. Unfortunately, several methodological challenges (discussed in the above response to reviewer 2) limit our ability to provide these data within a reasonable timeframe and without additional funding. We therefore hope it will be possible to accept the manuscript with the data currently included.

Reviewer #3: None

**Part III – Minor Issues: Editorial and Data Presentation Modifications**
Please use this section for editorial suggestions as well as relatively minor modifications of existing data that would enhance clarity.

Reviewer #1: See Part I

Reviewer #2: It would help readability if the authors included the identity of each antibody used in confocal images within the figure panel rather than only in the legend.

**Response:** Thank you for this suggestion. We agree that this makes the figures much easier to read and have adjusted all confocal images accordingly.

Reviewer #3: Minor points:

Figure 1B-C - visualizing a decreasing parameter (binding) as an increase in "Loss of binding" is not optimal. Why not plot "Residual binding" as a falling curve?. Its more logical. And why not plot the average residual binding at each timepoint a points on a curve (as in figure 4)?

**Response:** Thank you for this suggestion. We agree that this makes the figure much easier to read and have adjusted it as suggested.

Figure 3. Infection of ASK cells. There are esterase inhibitors available that coud be tested for activity and effect in vitro. Any particular reason this was not included?

**Response:** Thank you for pointing this out. We agree that it would be very interesting to assess the effect of administering an esterase inhibitor to infected cells and did indeed try to do so. Because the viral esterase also has potential to affect viral entry (although not shown for ISAV), we would need to start inhibiting the esterase after viral entry, but before inhibition of binding becomes prominent. Moreover, because the inhibition develops gradually, we would need to continue over a relatively long time-period, probably from 24-48 h.p.i. However, we have found it challenging to find an esterase inhibitor that is well-tolerated by the ASK cells for a 24-hour period at concentrations that also efficiently inhibit esterase activity. Because this approach would still not distinguish between the effect of inhibiting the viral esterase and the considerable endogeneous esterase activity in ASK cells, we have chosen not pursued it further.

Fig S2 D Align subscripts (skewed to the right on the screen version but looks better when printed)

**Response:** Thank you for making us aware of the misalignment. We have now corrected it.
